# Gut barrier-microbiota crosstalk in sepsis: from pathogenesis to potential therapies

**DOI:** 10.3389/fimmu.2026.1865498

**Published:** 2026-06-18

**Authors:** Lingshuai Meng, Yingjie Liu, Nana Wang, Tiegang Li, Yu Wang, Mandi Li

**Affiliations:** 1 Department of Emergency Medicine, Shengjing Hospital of China Medical University, Shenyang, China; 2 Department of Clinical Trial Unit, Shengjing Hospital of China Medical University, Shenyang, China; 3 School of Mechanical Engineering, Shenyang Jianzhu University, Shenyang, China

**Keywords:** gut microbiota, inflammatory response, intestinal epithelial barrier, medical treatment, sepsis

## Abstract

Sepsis is a systemic inflammatory response syndrome triggered by infection, frequently complicated by severe organ dysfunction and high mortality rates. Recent studies of intestinal epithelial function and the gut microbiota have highlighted their pivotal roles in the pathogenesis of sepsis. However, the precise mechanisms governing the interaction between the intestinal epithelium and gut microbiota, and how this interaction drives sepsis progression, still need to be elucidated. In this review, the functions of the intestinal epithelial barrier are first outlined, and the clinical significance of its altered permeability during sepsis is highlighted. Then, the physiological roles of the gut microbiota are further explored, detailing how dysbiosis and microbial metabolites influence disease progression and trigger both localized and systemic immune responses. Based on this, a logical framework for gut-originated systemic inflammation is proposed, and the potential adverse effects of current clinical supportive therapies on intestinal integrity are further discussed. Finally, the emerging sepsis treatment strategies that target gut function are summarized, aiming to provide novel insights and therapeutic directions for clinical practice.

## 1 Introduction

Sepsis is a life-threatening organ dysfunction caused by a dysregulated host response to infection (1). Globally, the mortality rate of sepsis is 10-20%, which sharply increases to 40-50% when it progresses to septic shock (2, 3), accounting for millions of deaths annually. Screening tools such as the Sequential Organ Failure Assessment (SOFA) and the Systemic Inflammatory Response Syndrome (SIRS) criteria are now widely used in clinical practice for sepsis identification (4). However, the underlying pathophysiological mechanisms remain not fully elucidated, and optimal treatment strategies are still under investigation.

The dysregulated host response in sepsis could be pathophysiologically characterized by the coexistence of inflammatory response and immunosuppression, which are jointly regulated by various pathogenic factors, host characteristics, and environmental factors (5). Traditionally, the direct attack of pathogens on the immune system and abnormal immune responses are usually the main focus of sepsis research. Although not explicitly mentioned in the Sepsis-3 international consensus (1), the gut, as the largest peripheral immune organ in the human body (6), has been widely recognized as a key factor, since its dysfunction might trigger and sustain systemic inflammation (7). Growing evidence indicates that disruption of the intestinal barrier and dysbiosis might not only increase susceptibility to sepsis (8, 9) but also worsen prognosis by exacerbating inflammatory response and multiple organ failure (10).

Indeed, gut microbiota dysbiosis could impair the intestinal epithelial barrier (IEB) (11). During this process, pathogen-associated molecular patterns (PAMPs, such as lipopolysaccharide (LPS)) derived from the microbiota and damage-associated molecular patterns (DAMPs) from injured host cells might be released in large quantities. Recognized by the innate immune system, they would disrupt normal immune homeostasis, thereby eliciting strong local and even systemic inflammatory and immunosuppressive responses (12). Therefore, gut microbiota, the IEB, and the local or systemic immune system might constitute a tightly interacting functional triad. The disruption of homeostasis within this triad can be considered one of the pathological bases driving the progression of sepsis to critical states (13).

Accordingly, in this review, the crucial function of the IEB and its damage mechanisms in sepsis are first elucidated, with a focus on the key role of gut microbiota dysbiosis in this process. On this basis, the resulting local immune homeostasis imbalance in the gut, and how this local disruption extends to and exacerbates systemic inflammation and immunosuppression, is analyzed. Subsequently, by integrating the above aspects, the mechanisms underlying gut-originated sepsis are systematically elucidated. Then, based on this mechanistic framework, how the concurrent damage to the gut microbiota and the IEB in clinical settings drives the progression of sepsis is explored. Finally, current therapeutic strategies for sepsis that target intestinal function are described.

## 2 Literature search strategy

A comprehensive search of PubMed was conducted using the terms gut barrier, intestinal barrier, intestinal permeability, gut microbiota, microbiome, intestinal flora, sepsis, septic shock, therapy, treatment, intervention, and therapeutic strategy for studies published up to March 2026. Additionally, the reference lists of selected articles were manually reviewed. For traditional Chinese medicine interventions specifically, a search of ClinicalTrials.gov (https://clinicaltrials.gov/) was performed to identify ongoing or completed clinical trials.

## 3 Crucial role and dysfunction of intestinal epithelial barrier in sepsis

The intestinal barrier can be defined as a multi-layered defense system, primarily consisting of the physical barrier (the mucus layer and intestinal epithelial cells), the biological barrier (commensal gut microbiota), and the immune barrier (lamina propria immune cells). In this section, research related to the IEB, composed of intestinal epithelial cells and mucus, in the context of sepsis is discussed.

### 3.1 Overview of structure and function of intestinal epithelial barrier

**Figure 1:**
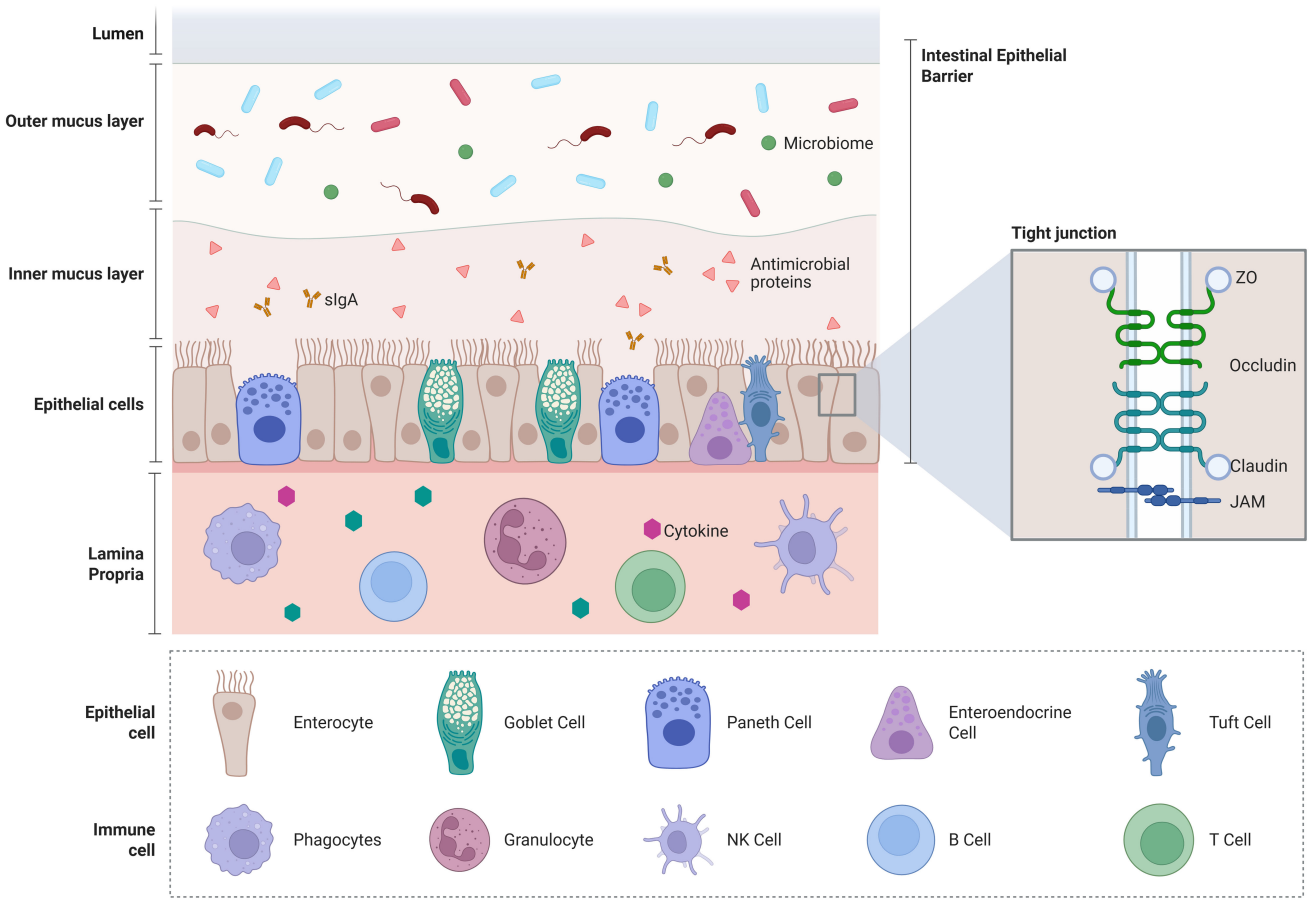
Structure of the intestinal barrier when taking the small intestine as an example. The intestinal barrier is a multi-layered composite structure. The intestinal epithelium and its tight junctions constitute the primary physicochemical barrier. The epithelium is primarily composed of five functionally specialized cell types, which are absorptive enterocytes, mucus-secreting goblet cells, IL-25-secreting tuft cells, hormone-secreting enteroendocrine cells, and antimicrobial peptide-secreting Paneth cells (14, 15). The lamina propria beneath the epithelium serves as the primary site of mucosal immunity, rich in innate immune cells (e.g., macrophages, dendritic cells, granulocytes, natural killer (NK) cells) and adaptive immune cells (e.g., T lymphocytes, B lymphocytes, and plasma cells). The permeability of the intestinal epithelium is dynamically regulated by intercellular tight junctions. Their crucial molecular components include: pore-forming transmembrane proteins (e.g., Claudin, Occludin), cell adhesion-mediating junctional adhesion molecules (JAMs), and intracellular scaffolding proteins (e.g., Zonula Occludens (ZO)) that anchor the aforementioned proteins to the cytoskeleton. Created in BioRender. ll, L. (2026) https://BioRender.com/3e3qmc2. Adapted from IIwasaki, A. (2026) BioRender.

The IEB is the primary physical structure of the intestinal barrier, consisting from top to bottom of the intestinal mucus layer and a single layer of intestinal epithelial cells. Intestinal epithelial cells can be divided into five main lineages based on their functions (14, 15), as shown in **Figure 1**. The lamina propria, located beneath the epithelium, is rich in immune cells such as dendritic cells, macrophages, and lymphocytes, constituting the robust immune barrier of the intestine. The integrity of the IEB would depend on the tight junctions between epithelial cells, which form dynamic interactions with the gut microbiota to jointly maintain selective permeability, ensuring nutrient absorption while blocking harmful substances (16).

Intestinal mucus is a gelatinous substance secreted by goblet cells and could provide a habitat for the gut microbiota. The mucus layer can be considered as the primary physical barrier, while the gut microbiota contributes to the first biological defense line against pathogens (17). The main components of mucus include water, mucins, lipids, and other proteins. Mucus plays a critical role in both innate and adaptive immunity as it is rich in antimicrobial peptides and immunoglobulin A (18). Mucus organization varies along the gastrointestinal tract: the small intestine has a single, loose layer that permits diffusion of digestive enzymes and nutrient absorption; the large intestine possesses a two-layered mucus structure, with an inner dense, essentially sterile layer that separates bacteria from the epithelium, and an outer layer that harbors commensal bacteria, including short-chain fatty acids (SCFAs) producers (19, 20). Furthermore, the gut microbiota could regulate mucus secretion and degrade mucus for use as a nutrient source (21). Through this interaction, intestinal mucus both provides the necessary physical separation for host-microbiota symbiosis and modulates key biological interactions via its selective filtering function, thereby might playing a pivotal role in maintaining intestinal homeostasis.

Beneath the mucus layer, the single-layer columnar epithelium constitutes a critical interface between the internal and external environments. Its integrity rests on the junctional complex at the apical lateral membrane, which comprises tight junctions, adherens junctions, and desmosomes (17). Among these, tight junctions are now regarded as the core structures regulating paracellular permeability, allowing only small molecules such as water and ions to selectively pass through under physiological conditions (21). Tight junctions are now defined as complex protein networks. The Claudin family might serves as the molecular basis for forming selective channels or barriers (22, 23). Occludin contributes to structural stabilization and regulation (24, 25). And intracellular scaffold proteins (e.g., ZO-1, ZO-2) anchor the entire network to the cytoskeleton (26). Zonulin has emerged as an important endogenous permeability regulator. Animal models and *in vitro* studies have shown that environmental triggers such as gluten can induce zonulin release, leading to tight junction disassembly and increased permeability via specific signaling pathways (27, 28). Commensal microbiota enhance barrier integrity partly through metabolites such as SCFAs, which upregulate tight junction proteins and promote epithelial restitution (29) (see Section 4 for details). Once the integrity of the IEB is compromised, excessive translocation of food antigens and microbial antigens would occur, leading to aberrant immune responses. This contributes to local intestinal inflammation and might be closely associated with the pathogenesis of various systemic diseases, such as diabetes, obesity, and metabolic dysfunction-associated steatotic liver disease(MASLD) (30).

### 3.2 Intestinal epithelial barrier disruption and its impact on intestinal permeability and local microenvironment

As previously described, the primary function of the IEB is suggested as to maintain stable selective permeability. In sepsis, the function is disrupted, directly leading to a pathological increase in intestinal permeability. This allows the translocation of luminal antigens into the lamina propria, thereby triggering an abnormal local immune response.

Under normal physiological conditions, an IEB effectively can separate the abundant gut microbiota in the intestinal lumen from immune cells in the lamina propria, maintaining a state of mutualistic homeostasis (31). However, during the pathological process of sepsis, multiple mechanisms collectively may lead to intestinal epithelial barrier dysfunction, with the most direct pathological consequence being an increase in intestinal permeability. This allows pathogens and their products (such as LPS and flagellin) in the intestinal lumen to translocate across the epithelial layer into the lamina propria and the portal circulation (17). In severe cases, viable bacteria from the gut may also translocate, leading to infections in vital organs in the mouse model of sepsis (32) and resulting in multiple organ dysfunction syndrome (MODS).

Intestinal epithelial barrier injury itself also directly exacerbates the deterioration of the local intestinal microenvironment. According to animal experiments, severe cellular stress may shifts epithelial cell death from orderly apoptosis to more intense necroptosis, releasing large amounts of DAMPs into the lamina propria (33, 34). Meanwhile, intestinal epithelial barrier injury would disrupt gut microbiota homeostasis, and the reduction of beneficial microbial metabolites (such as SCFAs) further impairs their supportive effects on epithelial cell maintenance and barrier repair (29). (35), thereby creating a self-amplifying vicious cycle locally.

### 3.3 Mechanisms of sepsis-induced intestinal epithelial barrier injury

The fundamental driver of the aforementioned vicious cycle of increased intestinal permeability and local microenvironment deterioration is the multiple hits of sepsis itself on the intestinal epithelial barrier. During sepsis, multiple pathogenic mechanisms converge to cause disruption of tight junction structures and a pathological increase in intestinal permeability. Underlying this process is the complete breakdown of the finely regulated tight junction protein network. Several animal studies have confirmed that upregulation of Claudin-2, which forms selective channels, is a key factor exacerbating gut leakage. In a mouse model of sepsis, Claudin-2 knockout significantly improved survival, suggesting that Claudin-2-mediated pore formation is an important mechanism aggravating the disease (36). In contrast, downregulation of key proteins that maintain barrier integrity, such as Claudin-1, Occludin, and the scaffold protein ZO-1, is directly associated with barrier injury. This view is strongly supported by interventional studies showing that various substances, including diosgenin, hesperetin, and Shengjiang San, effectively improve intestinal barrier function and survival outcomes in septic animals by specifically upregulating the expression of these tight junction proteins (37, 38, 39).

According to animal experiments and *in vitro* experiments, the imbalance of the tight junction protein network is rooted in the aberrant activation of more upstream signaling pathways. The mechanosensitive ion channel Piezo1 has been found to be abnormally active in sepsis, and the calcium influx it mediates triggers mitochondrial dysfunction, subsequently leading to intestinal epithelial cell apoptosis and physical disruption of tight junction structures (40). Deeper regulation originates from the intrinsic immune signaling pathways of intestinal epithelial cells. For example, the Myd88 pathway plays a central role in sensing gut microbiota dysbiosis signals. Evidence indicates that dysbiosis, through activating the intestinal epithelial Myd88 pathway, suppresses the key bile acid homeostasis regulatory axis (FXR/FGF15), thereby exacerbating systemic metabolic disorders while directly compromising intestinal barrier integrity (41).

Furthermore, gut microbiota dysbiosis itself may actively disrupt the epithelial barrier, thereby initiating systemic inflammation. Overgrowth of specific pathogenic bacteria, such as *Enterococcus faecalis*, can directly lead to a significant reduction in the expression of tight junction proteins ZO-1 and Occludin, accompanied by bacterial translocation to distal organs (e.g., the lungs), thereby escalating local infection into systemic sepsis, as demonstrated by animal experiments (32). Broader pathological models have provided evidence suggesting that bacterial translocation resulting from gastrointestinal injury is a direct trigger of sepsis-like systemic inflammatory response syndrome based on evidence from *in vivo* animal studies and human tissue pathology (42). Collectively, these studies establish the initiating and driving role of the microbiota-epithelial barrier axis in the pathogenesis of sepsis, in which gut dysbiosis, by altering intestinal permeability, may open the door for pathogens and their products to enter the systemic circulation, thereby potentially igniting an uncontrolled immune response.

## 4 Bidirectional modulation of gut microbiota and its metabolites in sepsis

In the occurrence and progression of sepsis, the imbalance of gut microbiota homeostasis and the injury of the intestinal epithelial barrier may be mutually causal and form a vicious cycle. Therefore, understanding the changes and functions of the gut microbiota and its metabolites is crucial for understanding the pathological mechanisms of sepsis. In this section, the composition and functions of the gut microbiota will be described first, with an emphasis on its potential bidirectional regulatory role in sepsis.

### 4.1 Composition and physiological functions of gut microbiota

The composition and functions of the gut microbiota vary among individuals living in different environments and with different habits. Most of these gut microbial communities can be classified into four major phyla, including *Actinobacteria*, *Firmicutes*, *Proteobacteria*, and *Bacteroidetes*, which together account for more than 98% of the total gut microbiota (43, 44). These microorganisms provide the host with absorbable nutrients, usable energy, and various metabolites with distinct functions (45). Factors such as climate change, dietary habits, and the host’s physiological state can influence the diversity and function of the gut microbiota (46–48) and are directly related to host’s health status.

In a healthy gut, the microbiota is characterized by a large population and high diversity. The gut microbial community is crucial for the development and regulation of the immune system, influencing the host’s susceptibility to infections and its ability to respond. To maintain a cooperative balance among microorganisms, the gut tends to establish a stable state of colonization resistance. This state can effectively inhibit pathogen invasion through at least three main mechanisms, namely, (1) niche competition between the normal microbiota and pathogens; (2) inhibition of pathogens by antimicrobial metabolites and bacteriocins produced by the normal microbiota; and (3) activation of the host immune defense system (43). When this balance is disrupted (e.g., due to antibiotic overuse or a weakened host immune system), previously harmless commensal gut bacteria may transform into opportunistic pathogens, leading to gut dysbiosis and even infection.

### 4.2 Interaction between gut dysbiosis and sepsis

Sepsis-associated dysbiosis is often regarded as a loss of obligate anaerobes and an expansion of potential pathogens (49, 50), which further aggravates barrier failure and fuels systemic inflammation. Given the complexity and significant individual variability of the gut microbiota, comprehensively revealing its relationship with sepsis remains challenging. Hence, two perspectives, the regulation of sepsis progression by the gut microbiota and the modulation of patients’ gut microbiota by sepsis, will be discussed in the following section.

The gut microbiota plays a significant regulatory role in the progression of sepsis. For instance, *Lentisphaeria* class, *Coprococcus2* genus, *Dialiste* genus, *Lachnospiraceae* UCG004 genus, *Victivallales* order, and *Lentisphaerae* phylum show positive effects in defending against the progression of sepsis, whereas *Clostridiaceae1* family, *Eubacterium eligens* genus, *Gordonibacter* genus, *Lachnospiraceae* ND3007 genus, and *Ruminococcaceae* UCG011 genus show negative effects through bioinformatics analysis (51). Protective microbiota may function through glutamate and lipid metabolism pathways, while harmful microbiota may influence sepsis progression via pathways such as lysophospholipase activity and inflammatory response regulation (51). Furthermore, studies have shown that gut microbiota detrimental to sepsis progression may affect the causal pathway of sepsis onset through C-reactive protein (CRP)-mediated effects, indicating that CRP may play a mediating role in the microbiota-sepsis relationship by bioinformatics analysis method (52). Additionally, under severe microbiota imbalance, certain opportunistic pathogens may directly participate in the initiation of sepsis. According to a clinical case report, *Bacteroides*, one of the common genus in the gut, can translocate when the intestinal barrier is compromised, thereby possibly becoming one of the pathogens responsible for sepsis (53).

In patients with sepsis, the gut microbiota exhibits characteristic dysregulation, typically characterized by a decrease in the abundance of anaerobic commensals (49) and an increase in the abundance of potential pathogens (50). This dysregulation, along with associated metabolic abnormalities, can further exacerbate disease progression by producing harmful substances or aberrantly activating immune responses (43). At the same time, the diversity of the gut microbiota is significantly reduced, while the abundance of opportunistic pathogens is greatly increased (54). LPS released by these overgrown Gram-positive bacteria may activate local immune responses in the gut. Furthermore, following bacterial translocation into the bloodstream, it is understood that these LPSs act as potent endotoxins, with the potential to activate signaling pathways such as TLR4, thereby exacerbating a systemic cytokine storm and consequently triggering or aggravating sepsis.

Specifically, changes in the gut microbiota during sepsis can be understood through their functional consequences, as summarized in **Table 1**. This functional perspective integrates the current physiological function classification of the gut microbiota (55) and focuses on how the presence or absence of these functions drives the progression of sepsis. Commensal taxa such as *Faecalibacterium*, *Bacteroides*, *Bifidobacterium*, *Blautia*, and *Ruminococcus* are primary producers of SCFAs (56, 57), and their decline during sepsis reduces SCFA availability, impairing energy supply to colonocytes, compromising barrier integrity, and diminishing anti-inflammatory signaling. Functionally, the loss of this metabolic output might drive barrier dysfunction. Taxa such as *Lactobacillus* and *Bacteroides* also contribute to barrier protection and immunomodulation, and their decrease reduces competitive exclusion of pathogens, though this effect is context-dependent (57, 58). Conversely, opportunistic pathogens including *Streptococcus*, *Enterococcus*, *Escherichia coli*, and *Staphylococcus* increase during sepsis, contributing to disease progression through LPS production, TLR4 activation, and translocation across the compromised barrier (54, 56). Hospital-acquired pathogens such as *Stenotrophomonas* and drug-resistant *E. coli* also proliferate, driven by medical interventions (56, 57), posing dual risks of antibiotic resistance gene carriage and superinfection. At the phylum level, the decrease in *Firmicutes* and increase in *Proteobacteria* reflect a functional transition from an SCFA-dominant, anti-inflammatory environment to an LPS-dominant, pro-inflammatory state, a shift more clinically relevant than the identity of individual taxa involved (56, 59).

**Table 1:** Functional shifts of the gut microbiota during sepsis.

Functional category	Representative taxa	Trends	Functional consequence	Ref
SCFA producers	*Bacteroides, Bifidobacterium, Faecalibacterium, Blautia, Ruminococcus*	*Decreased*	Reduced butyrate/acetate/propionate, impaired barrier integrity, reduced anti-inflammatory signaling, and reduced energy supply to colonocytes	(56, 57)
Barrier protectors	*Lactobacillus, Bacteroides*	Generally decreased	Reduced competitive exclusion, reduced immunomodulation (context-dependent)	(57, 58)
LPS/TLR4 activators	*Streptococcus, Enterococcus, Staphylococcus*	Increased	Increased LPS burden, TLR4 activation, pro-inflammatory cytokine storm, translocation risk	(54) (56)
Antibiotic-resistant/nosocomial	*Enterococcus, Escherichia Group, Stenotrophomonas, Escherichia coli*	Increased	Carriage of resistance genes, risk of hospital-acquired infection	(54) (56)
Phylum-level shift	*Firmicutes*	Decreased	Functional transition from SCFA-dominant (anti-inflammatory) to LPS-dominant (pro-inflammatory) metabolic environment	(56) (59)
	*Proteobacteria*	Markedly Increased		

### 4.3 Bidirectional relationship between the gut-liver axis and sepsis

Approximately 70% of the blood supply to the liver comes from the intestines. This anatomical arrangement appears to position the liver as a kind of filter for the portal venous circulation, meaning that substances derived from the gut, including nutrients, microbial metabolites, and toxins, may pass through the liver before entering the systemic circulation (60) At the same time, bile acids secreted by the liver are discharged into the intestinal lumen via the biliary tract. This bidirectional anatomical and functional interaction is commonly referred to as the gut-liver axis (61). Based on this, bile acids secreted by the liver can be further converted into secondary bile acids by the gut microbiota. Therefore, it seems plausible that an imbalance in the gut microbiota might contribute to the development of systemic sepsis, possibly by overactivating immune responses in the liver. Conversely, liver injury resulting from septic shock may lead to alterations in bile acid secretion, which in turn could further exacerbate gut microbiota imbalances and intestinal dysfunction.

First, an imbalance in the gut microbiota may lead to increased intestinal permeability, which could promote bacterial translocation. This, in turn, might push the liver beyond its immune response threshold, potentially triggering systemic inflammation and possibly progressing to sepsis. For example, relevant literature suggests that in critically ill patients, gut microbiota imbalance may contribute to LPS-induced increases in intestinal permeability, thereby facilitating the translocation of bacterial products to the liver (62). In patients with cirrhosis, an increased risk of bacterial infection appears to arise from both gut microbiota imbalance and loss of intestinal integrity, which may make them more likely to transition from acute liver injury to sepsis (63). The molecular basis of this mechanism may involve the activation of hepatic immune signaling pathways (such as TNF-α, Toll-like receptor 4, and NF-κB), which could activate Kupffer cells in the liver. This activation may allow gut-derived endotoxins to exert pro-inflammatory effects within the liver and potentially lead to hepatocyte apoptosis and necrosis (64, 60).

On the other hand, liver injury resulting from sepsis may also affect the composition and abundance of the gut microbiota. Sepsis is often accompanied by manifestations of shock or the pre-shock stage, which may lead to hepatic hypoxia and hypoperfusion. This, together with the deleterious effects of endotoxins and inflammatory cytokines, may contribute to impairments in the uptake, transport, and secretion of bile acids by hepatocytes (65). This could manifest as initially normal plasma total bile acid levels in pediatric patients during the early stages, even though the composition of individual bile acids may already be markedly disturbed (for example, increases in certain primary bile acids and the secondary bile acid TDCA, alongside decreases in other secondary bile acids such as DCA and UDCA). These alterations appear to be even more pronounced in cases of sepsis-induced cholestasis (66). Bile acids are molecules with antimicrobial activity. Changes in their concentration and composition may therefore directly influence the structure of the gut microbiota. For instance, animal studies suggest that certain bacteria capable of tolerating or metabolizing bile acids (such as *Escherichia/Shigella*) may overgrow, while others (such as *Bacteroides*) may be suppressed, potentially leading to an imbalance in the gut microbiota (67).

### 4.4 Immunomodulatory function of gut microbiota metabolites

The gut microbiota generates a variety of metabolites through different pathways. These substances tend to play important roles in host energy metabolism, nutrient absorption, and protein synthesis. They can also regulate intestinal mucosal barrier function and could influence the immune system. Metabolites produced by the gut microbiota include those that are generated by the host but secondarily synthesized by the microbiota, such as secondary bile acids and diet-derived metabolites, as well as products synthesized by the gut microbiota on their own, such as SCFAs. Among these, SCFAs and secondary bile acids are two representative metabolites that are present at high concentrations in the gut and exert relatively critical functional roles.

SCFAs, as crucial metabolites produced by the gut microbiota from indigestible dietary fiber, possess multiple physiological functions. They regulate local intestinal immune responses and maintain intestinal pH homeostasis and are essential for preserving gut microenvironment stability and systemic immune balance through pathways such as influencing bile acid metabolism. SCFAs mainly include butyrate, propionate, and acetate. SCFAs can be absorbed by the human body through the intestinal wall, with nearly 90% of them located in the gut and the remaining distributed in other parts of the body (68). When SCFAs regulate intestinal pH, they impair the synthetic functions of invading bacteria, thereby affecting their growth and reproduction (69) and inhibiting the expression of virulence genes in pathogens (43).

Within the intestine, SCFAs play an important role in intestinal immunity. For example, in the intestines of antibiotic-treated mice, SCFAs can enhance the secretion of IgA (70), which is the most abundantly produced immunoglobulin in the gut and is primarily synthesized by B cells and plasma cells in the intestinal mucosal tissue. Additionally, SCFAs promote the differentiation of regulatory T cells (Tregs) and exert broad anti-inflammatory effects, thereby effectively suppressing excessive immune responses against gut commensal microorganisms (6).

In addition, the immunomodulatory effects of SCFAs can be achieved through receptor-dependent mechanisms and the inhibition of histone deacetylases. Regarding the receptor-dependent mechanism, SCFAs might regulate immune responses by modulating signaling pathways. For example, by specifically binding to and activating G protein-coupled receptors on the cell membrane, which are present on intestinal epithelial cells and certain immune cells (such as neutrophils and macrophages) (71), thereby triggering rapid intracellular signal transduction and regulating immune, metabolic, and barrier functions. Regarding the mechanism involving histone deacetylase inhibition, butyrate can directly enter the nuclei of immune cells and intestinal epithelial cells. Animal and *in vitro* experiments have shown that, by increasing the level of histone acetylation, it persistently upregulates the synthesis of anti-inflammatory factors, tight junction proteins, and mucins, while simultaneously inhibiting the transcription of genes associated with key pro-inflammatory pathways (such as NF-κB) (71).

After being absorbed from the gut and entering systemic circulation, SCFAs might also be crucial in the host’s immunity and metabolism. For example, SCFAs can act on peripheral immune cells (such as macrophages and lymphocytes), thereby suppressing excessive systemic inflammatory responses. Specifically, SCFAs might influence collagen metabolism and degradation by regulating macrophage polarization, this can be seen from animal experiment analyzing results; for instance, butyrate exerts its anti-inflammatory effects through this process (72). In addition, SCFAs promote the differentiation of regulatory T cells and affect T cell metabolism and signal transduction (72).

In addition to SCFAs, secondary bile acids represent another important class of microbiota-derived metabolites. Bile acids are normal metabolites in the intestinal lumen and are essential for the digestion and absorption of lipids, as well as for the uptake of cholesterol and fat-soluble vitamins. Most bile acids are reabsorbed via the enterohepatic circulation, while 5%-10% of the unabsorbed bile acids serve as substrates for gut microbes and are converted into secondary bile acids (71). For example, Clostridium species within the phylum Firmicutes are capable of synthesizing secondary bile acids (71). Although they are metabolites of the gut microbiota, secondary bile acids might regulate lipid signaling pathways and the immune system through their receptors TGR5, FXR, and PXR, as demonstrated by animal experiments and *in vitro* cell experiments (71). With regard to the immune system, secondary bile acids exert favorable anti-inflammatory effects at low concentrations by reducing the levels of pro-inflammatory cytokines according to mouse experiments and *in vitro* experiments (74). In contrast, when present at high concentrations in the gut, secondary bile acids can induce oxidative/nitrosative stress, DNA damage, apoptosis, and mutations, and may even trigger inflammation (71, 75, 76).

### 4.5 Metabolite deficiency or imbalance exacerbates sepsis pathology

Currently, in research on gut microbiota associated with sepsis, scholars have begun to recognize that the deficiency of gut-derived SCFAs in sepsis may accelerate disease progression, and have started investigating the mechanisms of SCFAs in sepsis. Previous studies have largely focused on the impact of butyrate on sepsis, as butyrate is strongly associated with immunity. However, current research indicates that propionate and acetate also have crucial implications in sepsis.

In mouse experiments, supplementation with SCFAs effectively restores gut microbiota homeostasis, enhances the expression of intestinal tight junction proteins, and activates the NLRP6 inflammasome in colonic tissue, thereby reducing the severity of sepsis-associated encephalopathy (77). In mouse models of sepsis-associated acute kidney injury, SCFA (particularly butyrate) levels are significantly reduced. Butyrate also alleviates NLRP3 inflammasome-mediated pyroptosis and inhibits the activation of the STING-GSDMD signaling pathway, thereby exerting a protective effect against sepsis-associated acute kidney injury (78). Furthermore, according to animal experiments, butyrate suppresses the expression of pro-inflammatory cytokines, promotes mucus secretion, and maintains the expression levels of immunoglobulin A and mucin-producing goblet cells (79). This provides crucial support for intestinal barrier function and immune function. Additionally, animal studies show that SCFAs improve the survival rate of septic mice through immune regulation (i.e., by reducing the release of mature IL-1β and inhibiting neutrophil extracellular trap formation to suppress CASP5-dependent pyroptosis) and metabolic restoration (i.e., by enhancing glycerophospholipid flux) (72). Moreover, propionate exhibits the most significant metabolic activity in peripheral plasma cells. Bioinformatics analysis and *in vitro* experiments revealed that it participates in the immune regulation of sepsis by regulating the IRF4/ARID3A/FOXO4 transcriptional network, mediating novel cell communication pathways such as BMP8B-BMPR2, and influencing antibacterial immune responses and fatty acid metabolism (80). Regarding the mechanism of acetate, it has been found in preclinical experiment that gut microbiota-derived SCFAs delay neutrophil apoptosis via the FABP4-endoplasmic reticulum stress axis in sepsis-induced lung injury, thereby exacerbating inflammatory factor-mediated lung epithelial damage (81).

Clinically, the levels of gut-derived SCFAs in sepsis patients during their ICU stay are generally decreased in observational study, suggesting that lower butyrate concentrations may indicate a higher mortality rate (56). In addition, SCFAs may influence gut microbiota colonization status and directly improve intestinal epithelial barrier function. For example, butyrate reduces group B Streptococcus-induced cell death, invasive capacity, and trans-barrier translocation ability *in vitro* and animal experiment (82).

Studies on sepsis and secondary bile acids indicate that secondary bile acid concentrations are reduced in patients with sepsis, which may be associated with gut microbiota dysbiosis during sepsis. However, it remains unclear whether this change directly affects the pathological progression of sepsis (83, 56).

## 5 Characteristics and imbalance of intestinal immune responses in sepsis

The gut is the largest peripheral immune organ in the human body and serves as a key site for regulating systemic immunity. Intestinal epithelial barrier dysfunction and gut microbiota dysbiosis may serve as major potential triggers of sepsis, while the gut itself possesses the capacity for early recognition and regulation, typically initiating a localized inflammatory response first. As a systemic inflammatory condition, gut-derived sepsis may often begin with the uncontrolled spread of local intestinal inflammation. In this section, the mechanisms underlying the formation of local intestinal inflammation and its progression toward systemic inflammatory imbalance will be elucidated.

### 5.1 Recognition of gut-derived danger signals and initiation of immune responses

The deepest layer of the intestinal epithelial barrier, namely the lamina propria, is the primary site where immune cells accumulate. To maintain the dynamic balance of intestinal permeability, intestinal epithelial cells continuously monitor various substances within the intestinal lumen through a series of immune mechanisms. These substances include microorganisms and their metabolites, dietary nutrients, and immune signals.

During the development of sepsis associated with intestinal epithelial barrier dysfunction, translocated intestinal pathogens and their products can be sensed by immune cells and epithelial cells positioned at the front line of intestinal mucosal defense. These cells recognize pathogen-associated molecular patterns, such as LPS and flagellin, through Toll-like receptors expressed on their surfaces (17). This recognition event rapidly triggers downstream signal transduction pathways and induces the onset of inflammatory responses, constituting an important component of the host’s innate defense (84). However, in sepsis, due to cellular stress, injury, or exposure to enteric pathogens, the primary mechanism of intestinal epithelial cell death may shift from apoptosis, which depends on specific receptor-mediated signals, to necroptosis, which is a more violent form of cell death directly triggered by inflammatory signals (33). Once necroptosis occurs in intestinal epithelial cells, large amounts of cellular contents are released, including various damage-associated molecular patterns and other potent pro-inflammatory mediators (34). These substances can be recognized by innate immune cells, which promote the release of inflammatory cytokines and guide the differentiation of immune cells. Furthermore, necroptosis also affects adaptive immunity. Antigenic materials released by necrotic cells can be efficiently captured, processed, and presented to T cells by professional antigen-presenting cells, such as dendritic cells, thereby shaping and modulating the pattern of T cell immune responses (85). Therefore, reducing intestinal epithelial cell apoptosis and systemic cell apoptosis in sepsis is also one of the current research directions (86, 87).

### 5.2 Biphasic imbalance of systemic immune responses in sepsis

Building upon the danger signals that are systemically sensed and amplified as described above, the host may enter a characteristic stage of systemic immune response in sepsis, which is often marked by the coexistence of early excessive inflammation and subsequent immunosuppression.

During the patient’s hyperinflammatory phase, large amounts of DAMPs and PAMPs released by necrotic and apoptotic cells as well as by microorganisms excessively activate innate immune cells such as macrophages, dendritic cells, and neutrophils through various pattern recognition receptors, including Toll-like receptors and Fcγ receptors (88). This triggers a cytokine storm, leading to a sharp surge in the levels of pro-inflammatory cytokines such as IL-1β, IL-6, and TNF-α (89). In these cytokines further disrupt immune balance, i.e., they drive the polarization of CD4+ T cells toward the pro-inflammatory Th1 subset and suppress the function of anti-inflammatory Th2/Treg cells, breaking their homeostasis, as observed in a prospective observational study (90). At the same time, activated CD8+ T cells undergo massive proliferation and acquire potent cytotoxic activity in mouse experiments (91). In addition, the cytokine storm itself causes extensive pathological damage, particularly by inducing excessive production of nitric oxide, which significantly increases vascular permeability (92), thereby leading to distributive shock, tissue hypoxia, and coagulation dysfunction. Excessive levels of factors such as IL-1β can also cause persistent hyperactivation of macrophages, forming a positive feedback loop (89).

Subsequently, the host enters the immunosuppressive phase, characterized by a sustained state of immune paralysis. This phase is marked by extensive immune cell depletion and dysfunction, that is, widespread programmed apoptosis of both innate and adaptive immune cells occurs (e.g., massive death of mature neutrophils, CD4+ T cells, CD8+ T cells, and B cells) (89, 93, 94), while dysfunctional immature neutrophils expand and infiltrate tissues (95). At the same time, surviving cells fall into a state of paralysis or exhaustion, manifesting as hyporesponsiveness of T cells and severely impaired antibody production by B cells (89). As a result, the immune system fails to clear the primary infection and becomes highly susceptible to secondary nosocomial infections or reactivation of latent viruses (96).

## 6 From gut to systemic inflammation: integrated mechanisms and clinical implications

In sepsis, intestinal barrier dysfunction and gut microbiota dysbiosis may collectively drive local intestinal inflammation and participate in systemic pathological processes. Substantial evidence has been accumulated regarding each of these individual components. Thus, integrating these discrete elements into a clear and dynamic logical framework of gut-originated systemic inflammation is likely essential for a complete understanding of sepsis pathophysiology. In this section, an integrated model is constructed that may illustrate the cascade and amplification relationships among gut microbiota dysbiosis, barrier injury, immune activation, and systemic inflammatory responses. Subsequently, based on this model, how common clinical interventions may exert their effects by influencing the above intestinal mechanisms is explored, thereby potentially providing a theoretical basis for optimizing clinical management strategies.

### 6.1 Gut-originated vicious cycle and its role as a pathogenic framework for sepsis

**Figure 2:**
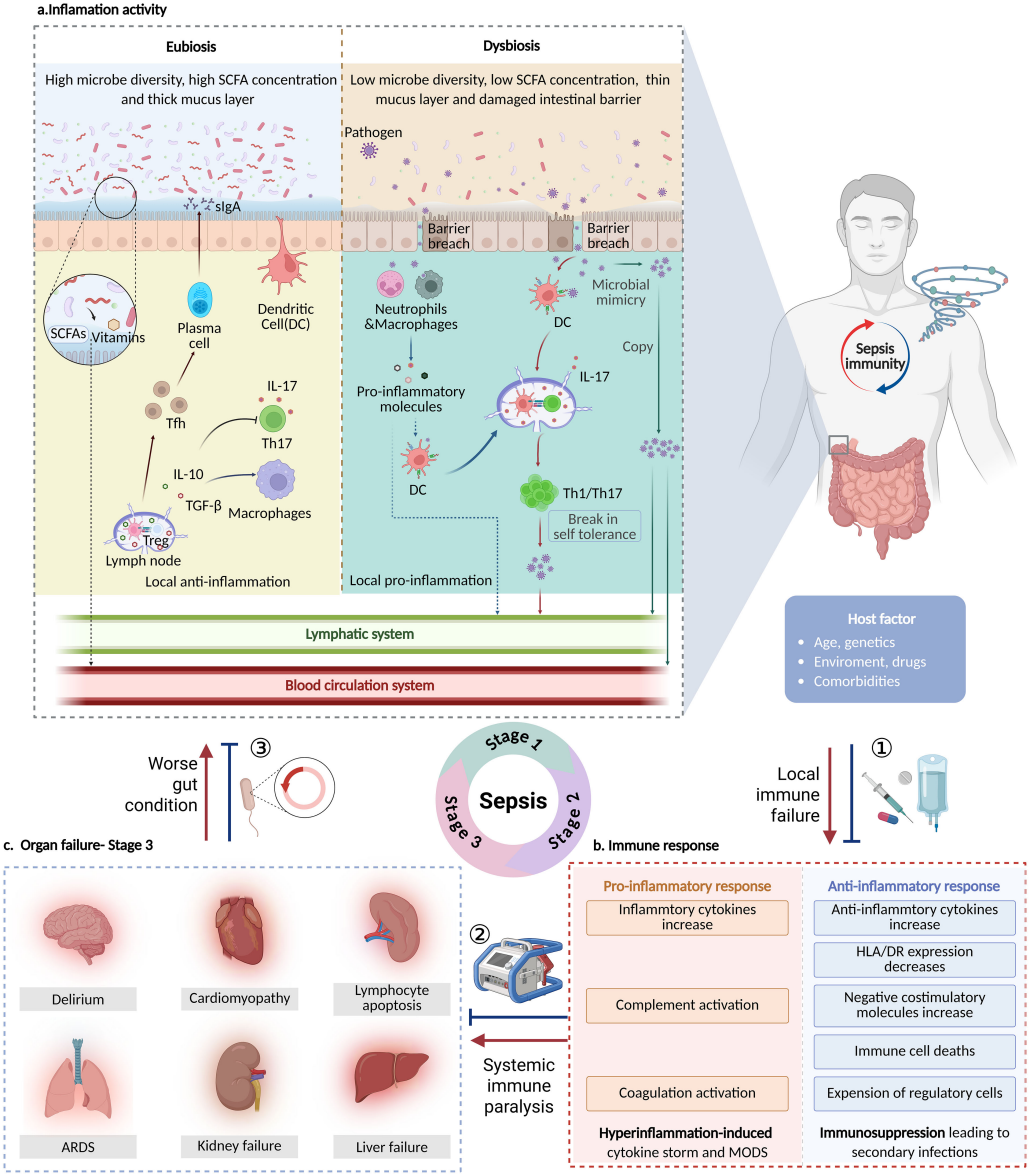
Gut-origin vicious cycle in sepsis pathogenesis. This schematic illustrates the proposed mechanistic link between gut dysfunction, systemic inflammation, and multi-organ failure. **(a)** Transition from intestinal eubiosis to dysbiosis and local immune activation. **(b)** Systemic immune consequences, featuring concurrent pro-inflammatory (cytokine storm) and compensatory anti-inflammatory (immunosuppression) responses. **(c)** Resultant organ failures and their feedback effects on intestinal integrity. These three stages will form a vicious cycle if there is no clinical treatment, as shown by red arrow direction. Appropriate treatments, as shown by the blue arrows, can be applied at different stages to slow down or even halt this vicious cycle. Key elements are labeled as indicated. Created in BioRender. ll, (L) (2026) https://BioRender.com/6rqpvtg.

To integrate the mechanisms discussed above, we propose a conceptual model of a self-perpetuating gut-originated vicious cycle that might drive the progression of sepsis, as illustrated in **Figure 2**. This model delineates three interconnected pathogenic stages.

Stage 1: Gut Homeostasis Disruption and Local Barrier Injury (**Figure 2a**). Under homeostatic conditions, gut microbiota diversity, adequate SCFAs, and an intact mucus layer may collectively maintain epithelial barrier function and immune stability. When external factors or host internal environment disturbances occur, gut homeostasis may be disrupted, potentially leading to a state of dysbiosis. This is often directly manifested by decreased microbial diversity, insufficient synthesis of SCFAs, and defects in the mucus layer. Together, these changes can erode the physical barriers of the intestinal epithelium, with the molecular mechanism being a significant downregulation of tight junction protein expression and disruption of their function.

Intestinal barrier injury exposes pathogens and their antigens, thereby potentially activating dendritic cells, neutrophils, and macrophages within the lamina propria. Upon activation, these immune cells may establish a pro-inflammatory microenvironment dominated by factors such as IL-17. This microenvironment drives Th1 and Th17 cell differentiation and breaks immune tolerance, thereby suppressing TGF-β mediated anti-inflammatory pathways. This local inflammatory environment may further compromises tight junction structures, thus forming a self-amplifying cycle of inflammation, barrier injury, and exacerbated inflammation. This positive feedback mechanism may dramatically amplifies local inflammatory damage and promotes the translocation of enteric pathogens into the systemic circulation via routes such as the portal vein, thereby potentially initiating systemic immune responses and thus leading to Stage 2.

Stage 2: Systemic Immune Dysregulation (**Figure 2b**). Uncontrolled local inflammation allows pathogenic bacteria and their products to enter the systemic circulation, triggering a robust sys may temic immune response. This stage often presents a dual and paradoxical immune status as described in Section 5.2. First, an intense pro-inflammatory response, including cytokine storm, complement and coagulation activation, can leads to extensive pathological damage. At the same time, as a consequence of compensation or overactivation, the host may enters a state of immune paralysis, characterized by immune cell depletion and functional suppression, resulting in loss of pathogen clearance capacity. This imbalance may be a key driver of multiple organ dysfunction.

Stage 3: Multiple Organ Failure and Feedback to the Gut (**Figure 2c**). The combined effects of systemic hyperinflammation and immune paralysis may lead to remote organ failure, manifested as acute respiratory distress syndrome (ARDS), liver and kidney failure, cardiomyopathy, and encephalopathy. Critically, this systemic damage, particularly high circulating levels of pro-inflammatory cytokines and the hemodynamic instability of shock, in turn causes further injury to the intestinal epithelium, thereby inducing apoptosis of intestinal epithelial cells and exacerbating the initial barrier dysfunction (97). This feedback loop, in which the systemic consequences of gut-derived inflammation subsequently aggravate the original intestinal injury, may establishes and perpetuates the vicious cycle of the model (98).

Model Considerations: It should be noted that although the above model begins with gut microbiota imbalance and impairment of the intestinal barrier function, depicting a vicious cycle in which gut microbiota translocation triggers sepsis and systemic multi-organ failure, which in turn further weakens intestinal function, the primary trigger of sepsis may not necessarily originate from gut microbiota translocation but could also be initiated by infections in other sites. In such cases, the starting point of the cycle may correspond to Stage 2 of the model. Nevertheless, once the septic state is established, if early and effective intervention is not implemented, the clinical course may still evolve into the aforementioned vicious cycle, subsequently leading to multi-organ failure and progressive decline in intestinal function, thereby perpetuating disease deterioration.

This conceptual model may provide a useful framework for understanding sepsis progression. Based on this model, potential therapeutic strategies could target key nodes of the vicious cycle as shown in **Figure 2**: ① preventing gut microbiota translocation and limiting inflammation spread by using anti-infective therapy (Stage 1→2, which corresponds to the early phase of sepsis); ② employing supportive measures to block progression to multi-organ failure (Stage 2→3, which corresponds to the established septic phase); and ③ maintaining gut homeostasis and barrier integrity through nutritional support and microbiota modulation (Stage 3→1, which corresponds to the post-sepsis period).

### 6.2 Clinical implication 1: preexisting intestinal dysfunction as a risk amplifier

Based on the gut-originated vicious cycle model presented in Section 6.1, it may be inferred that any preexisting intestinal barrier vulnerability or gut microbiota dysbiosis might significantly lower the threshold for a patient to withstand the insult of sepsis and may accelerate the progression toward potentially irreversible systemic inflammation and organ failure. Patients with chronic inflammatory or metabolic diseases that are closely associated with gut dysbiosis and increased intestinal permeability may exhibit a particularly elevated risk of sepsis susceptibility and adverse outcomes. This risk has been suggested by evidence from confirmed in a variety of clinical scenarios, with the underlying pathophysiological basis consistently often pointing to specific intestinal defects. Clinically actionable biomarkers of intestinal vulnerability (e.g., zonulin, fecal calprotectin, NLR, PLR, HALP,CRP) and metabolic dysregulation (HbA1c, fasting glucose, BMI) enable early risk stratification before sepsis onset (99–101).

For patients with inflammatory bowel disease (IBD), the condition itself often serves as a typical example of this mechanism. IBD is characterized by gut dysbiosis, local immune activation, and chronic intestinal barrier disruption (102). Consequently, when undergoing major surgeries such as total knee arthroplasty or cervical spinal fusion, IBD patients may exhibit a significantly higher risk of postoperative infections and sepsis compared to non-IBD patients (approximately 1.5-fold) (103, 104). Fecal calprotectin, elevated zonulin, and reduced microbial diversity can stratify IBD patients into high-risk subgroups for postoperative sepsis (105, 106, 59). This observation is consistent with the inference that the already compromised intestinal state in IBD patients may increases their susceptibility to sepsis.

In patients with obesity and metabolic syndrome, the intestinal environment exhibits characteristic alterations, including low-grade inflammation, increased intestinal permeability, and reduced microbial diversity or compositional dysbiosis (107). Clinical data are consistent with this pathological basis. Among septic patients admitted to the ICU, the mortality group had a higher mean BMI (30.33 ± 6.88) compared to the non-mortality group (27.13 ± 5.54) (100). Furthermore, indicators associated with type 2 diabetes, such as elevated fasting glucose, BMI≥25, and increased C-reactive protein levels, have been identified as independent predictors of postoperative sepsis (101).

It is worth noting that MASLD, as a typical manifestation of obesity and metabolic syndrome in the liver, may further amplifies this risk through the gut-liver axis mechanism. Patients with MASLD commonly exhibit gut microbiota dysbiosis, which continuously activates the gut-liver axis via pathways such as endotoxin translocation (108). The chronic gut-liver axis dysregulation may lead to a more critical illness course when MASLD patients develop sepsis. Studies have shown that compared with non-MASLD patients, MASLD patients with sepsis have significantly higher SOFA scores and APACHE II scores (109), suggesting more severe organ dysfunction, greater disease severity, and poorer clinical outcomes in the setting of sepsis. Preoperative MASLD screening (CT attenuation ≤40 HU) stratifies patients at highest risk of sepsis progression.

Due to this, diseases such as inflammatory bowel disease, obesity, and metabolic syndrome, through distinct pathways of chronic inflammation, metabolic dysregulation, and gut-liver axis dysfunction, may lead to a state of intestinal injury or preinjury. When such patients encounter the insult of sepsis, their already fragile gut homeostasis rapidly collapses, unleashing a more intense systemic inflammatory response, which ultimately manifests as a higher incidence of sepsis, possibly more severe organ damage, and poorer clinical outcomes. The implementation of prophylactic interventions prior to major surgeries and the development of individualized perioperative management plans can reduce the risk of sepsis in high-risk patients, minimize complications, and improve clinical outcomes (104).

### 6.3 Clinical implication 2: therapeutic paradox and gut protection

When treating high-risk patients with a fragile intestinal barrier, as described in Section 6.2, current standard supportive care for sepsis faces a critical paradox. Clinical measures designed to maintain circulatory and respiratory function may actually constitute a secondary insult to the patient’s already vulnerable intestinal ecosystem, thereby further promoting the aforementioned vicious cycle.

After patient admission, clinical management for sepsis and multiple organ failure typically includes antibiotics, fluid resuscitation, respiratory support, as well as other measures such as vasoactive agents and nutritional support (110, 111). However, these life-saving interventions may paradoxically induce or exacerbate intestinal dysfunction, manifesting as intestinal edema, diarrhea, and more severe dysbiosis, all of which are independently associated with increased mortality (112).

The mechanisms underlying this iatrogenic damage include the following. Most broad-spectrum antibiotics deplete gut commensals and increase the risk of intestinal infections (113, 114). Anti-anaerobic antibiotics may promote overgrowth of intestinal pathogens and weaken systemic immunity, subsequently increasing the risk of nosocomial infections and contributing to higher mortality in septic patients (115). Hemodynamic instability, including persistent hypoperfusion and ischemia-reperfusion injury, might damage the intestinal epithelial barrier and disrupt the integrity of tight junction structures (116). Vasoactive agents used in septic shock may induce intestinal dysmotility and intolerance to enteral nutrition (117), thereby affecting nutrient absorption and mucosal repair. Additionally, the timing and strategy of enteral nutrition themselves pose a challenge; inappropriate implementation may lead to intestinal atrophy, gut dysbiosis, and exacerbate multiple organ dysfunction syndrome (118), whereas aggressive nutritional support before septic shock is fully reversed may precipitate mesenteric ischemia (112).

This treatment paradox may indicate that in sepsis management, the functional status of the gut should not be merely a secondary consideration but rather a central therapeutic focus, as its further deterioration can directly lead to an increased risk of death. The risk may be particularly pronounced in patients with underlying conditions such as obesity, inflammatory bowel disease, or diabetes. Their inherently fragile intestinal ecosystem may have a significantly reduced compensatory capacity against such iatrogenic insults, resulting in a disproportionately increased risk of severe intestinal dysfunction, secondary infections, and poor outcomes. Therefore, clinical practice not only needs to recognize this paradox but also requires the prospective integration and implementation of active gut-protective strategies into standard sepsis bundle care.

## 7 Potential therapeutic strategies targeting the intestinal barrier and microbiota

From a pathophysiological perspective, immune response dysregulation and the resulting epithelial and coagulation dysfunction are the fundamental features of sepsis. Therefore, in addition to conventional supportive care, targeted therapies addressing key biological drivers have become a major focus of clinical research and development. Currently, various targeted agents such as anti-TNF monoclonal antibodies to correct excessive inflammation, recombinant human IL-7 to reverse immunosuppression, and recombinant human soluble thrombomodulin to modulate coagulation dysfunction have entered clinical trials (94).

**Figure 3:**
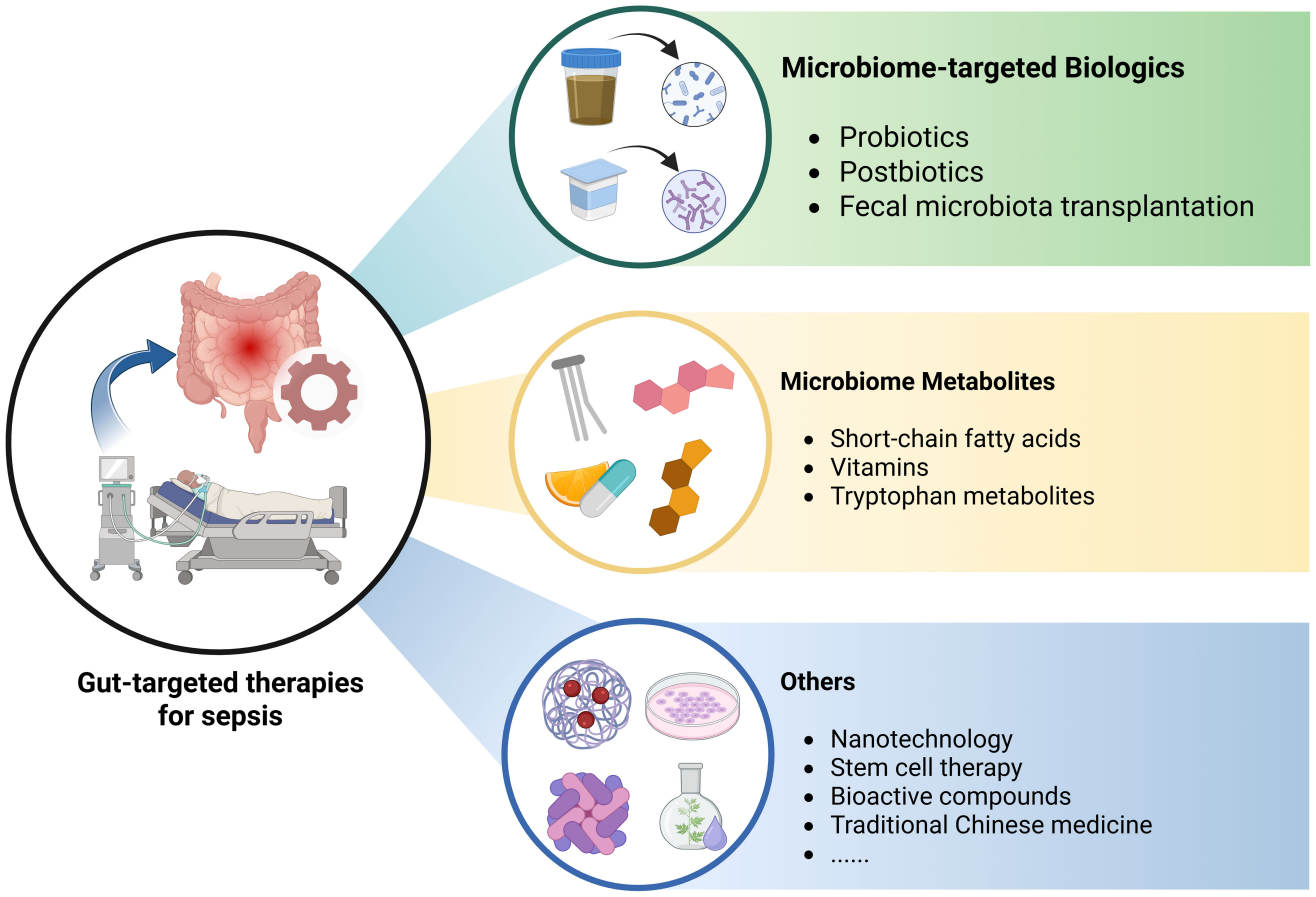
Therapeutic strategies targeting gut function in sepsis management. This image summarizes three main approaches aimed at improving gut function in sepsis treatment, based on human and animal studies. (1) Microbiome-targeted biologics: Primarily encompassing probiotics, postbiotics, and fecal microbiota transplantation. This approach has been validated in both human studies and animal experiments. (2) Microbiome-derived metabolites: SCFAs are recognized as key therapeutic agents. Other metabolites such as vitamins and tryptophan derivatives are also under investigation. This approach has only been investigated in animal trials, (3) Others: These include emerging technologies such as nanotechnology (for drug delivery via nanoparticles), stem cell therapy, and the application of specific bioactive compounds (commonly used in newborn sepsis treatment), along with traditional Chinese medicine. This approach has only been evaluated in animal trials. Created in BioRender. ll, L. (2026) https://BioRender.com/ccba5z0.

On the other hand, as mentioned above, gut microbiota dysbiosis and barrier damage may also contribute significantly to the pathogenesis of sepsis. Therefore, intervention strategies aimed at restoring intestinal microecological balance and enhancing intestinal mucosal barrier function have attracted considerable research interest. Current research in this field mainly focuses on areas such as microbiome-targeted biologics, microbial metabolites, and others, as shown in **Figure 3** and **Table 2**. However, as will be discussed below, these approaches vary widely in their evidence base, clinical maturity, and safety profiles. Critically, the risks associated with microbiome manipulation in septic patients, particularly those who are critically ill, remain underemphasized in the existing literature.

**Table 2:** Therapeutic interventions for gut permeability and microbiome in sepsis treatment.

Interventions	Description	Effects on gut function	Limitation	Reference	
				Experimental models	Human Studies
Microbiome-targeted Biologics					
Probiotics	Live microorganisms (typically bacteria)	Improvement of intestinal barrier function and immune response	Might promoting specific microbiome growth	(120–123) (143)	(144, 145)
Prebiotics/Postbiotics	Beneficial bacteria growth/consisting of inactivated microbial cells or their components			(126)	(124, 125)
Fecal microbiota transplantation	Transplantation of processed donor fecal microbiota into patients GI tract	Improvement of composition of gut microbiota	Variable clinical efficacy for critically ill patients	(73) (146) (147, 148)	(129)
Microbiome Metabolites					
SCFAs	Fermentation of dietary fiber by the gut microbiota	Improvement of gut function and recovery of organ injured due to sepsis	No clinical data, mainly in mouse experiments	(77) (79) (81) (82)	-
Vitamins	Produced by gut microbiota or found in fermented foods	Suppression of inflammation and reduction of skeletal muscle injury in sepsis		(131)	-
Tryptophan metabolites	Produced by gut microbiota via the indole pathway	Reduction of intestinal inflammation and adjustment of intestinal immune homeostasis by its derivative, indole-3-lactic acid.		(132)	-
Others					
Nanotechnology	Precision targeting of nanoparticle-based carriers	Nanoparticles for precise drug delivery to remodel gut microbiota with permeability and necroptosis	Biological safety and tolerance profile of nanoparticles	(87) (133) (134)	-
Stem cell therapy	Use stem cell to decrease sepsis-induced organ Injury	Restoring the gut microbiota to a normal state	Unclear key mechanism	(135)	-
Bioactive compounds	Endogenous bioactive constituents from human	Adjusting gut microbiota diversity and upregulating antimicrobial peptides	No clinical data	(86) (136)	-
TCM	Single active ingredients: Bioactive lead compounds of plant/herbal origin	Optimization on composition of gut microbiota, improvement of intestinal barrier function, decrease of inflammation and apoptosis and even increase of production of SCFAs.	No clinical data, mainly from animal experiments and *in vitro* studies.	(38) (149) (140, 141)	-
	Herbs and complex extracts: Medicinal plant itself, or non-single-component mixture extracted from it.			(138, 139)	-
	Chinese herbal formula: Fixed combination of multiple medicinal materials guided by TCM theory			(39) (121)	-

### 7.1 Microbiota-targeted biologics

During sepsis, the gut microbiota is prone to functional dysbiosis. Hence, protecting its diversity and enhancing its stability through microbiota-targeted biologics has become an important intervention strategy. However, when applying these biologics to critically ill patients, the potential benefits must be carefully weighed against the non-negligible risks. Specific approaches include the use of probiotics, prebiotics/postbiotics, and fecal microbiota transplantation, which also serve as important adjunctive means for regulating intestinal function in critically ill patients.

Probiotics prevent subsequent infections by introducing live microorganisms to restore the balance of the damaged microbiota (119). Their mechanisms include altering the composition and abundance of the microbiota and influencing the expression of tight junction proteins, as demonstrated in the animal experiments by *Saccharomyces boulardii (*120) and COF-based artificial microbiota (121). They also regulate pyroptosis and improve lung function via the gut-lung axis, such as the ΔPESI engineered *Escherichia coli* strain (122). Additionally, they promote intestinal stem cell regeneration and protect the intestinal barrier, as seen with the *Lactobacillus rhamnosus* GG strain (123). Additionally, prebiotics promote the growth of beneficial bacteria by providing nutrients (119), with human milk oligosaccharides being a typical example in human study considered an important prebiotic for the neonatal gut (124). Postbiotics refer to non-living microbial components or metabolites that are beneficial to the host (125). It has shown that pasteurized Weissella cibaria exerts its effects by improving the intestinal mucosal barrier, reversing dysbiosis, and increasing the abundance of anti-inflammatory bacteria in mouse experiments (126). Although these three types of interventions can increase microbial populations, their effects may be unstable and accompanied by side effects (127). Especially in critically ill patients, the use of probiotics carries a risk of lactobacillus or yeast bacteremia, and therefore should be used with caution in individuals with severe impairment of the intestinal barrier (128).

Fecal microbiota transplantation (FMT) restores the balance and diversity of the microbiome by transplanting fecal microorganisms from a healthy donor into the patient (73). When combined with antibiotic therapy, FMT shows better efficacy in treating sepsis than antibiotics alone in animal experiments (73). However, the therapeutic efficacy of FMT remains inconsistent in clinical studies (129). In critically ill patients, FMT may not only cause bacterial translocation due to donor microbiota and unpredictable serious complications (129) (such as transmission of multidrug-resistant organisms), but also poses procedure-related safety concerns. Currently, the application of FMT in the routine treatment of sepsis still requires further investigation.

### 7.2 Microbiome metabolites

With the deepening of research, the regulatory role of gut microbiota metabolites on the host immune system has been confirmed. Consequently, the mechanisms of action of typical metabolites represented by SCFAs, vitamins, and tryptophan in sepsis have become a research hotspot in recent years. Currently, studies on such metabolites, especially SCFAs, are mostly still at the animal experiment stage.

SCFAs are a class of organic fatty acids produced by the fermentation of dietary fiber by gut microbiota, primarily including acetate, propionate, and butyrate. As mentioned earlier, animal experiments show that they might serve as important protective agents in sepsis (77, 79, 81, 82). For this reason, whether through herbal medicine interventions or microbiota-targeted biotherapeutics (such as prebiotics and synbiotics), increasing SCFA levels has been regarded as an important effect indicator for improving intestinal barrier function and regulating often immunity. Although SCFAs have shown great potential in animal experiments, their direct translation into clinical applications faces significant obstacles, including their extremely short half-life, difficulty in reaching the colon via oral administration, and the unknown side effects that may be associated with locally high concentrations (130). Therefore, SCFA-based therapies are still in the proof-of-concept stage.

In addition to SCFAs, the functions of other metabolites such as vitamins and tryptophan in sepsis have also attracted attention. For example, studies have found that vitamin K1 may suppress NF-κB-mediated inflammatory responses and may ameliorate sepsis-associated skeletal muscle injury through upregulating SIRT1 and regulating protein metabolic balance (131). The tryptophan derivative indole-3-lactic acid contributes to immune regulation and intestinal barrier maintenance. Specifically, it may protect the intestinal barrier, reduces bacterial translocation, and alleviates clinical symptoms, thereby potentially offering a new perspective for sepsis intervention (132). However, these findings are currently derived primarily from *in vitro* or animal models. The definitive efficacy of vitamin K1 and indole-3-lactic acid in patients with sepsis, particularly their regulatory effects on intestinal function, still requires validation through high-quality clinical studies.

### 7.3 Others

In addition to the aforementioned approaches, some intervention methods are difficult to classify into traditional categories and thus are collectively listed as other means. Such methods identified in current investigations mainly include nanotechnology, stem cell therapy, bioactive compounds and traditional Chinese medicine(TCM). However, these approaches are still limited to animal trials.

Nanotechnology and stem cell therapy are cutting-edge strategies in the treatment of sepsis. Nanotechnology has attracted attention due to its ability to precisely deliver drugs, regulate programmed necrosis, and enhance anti-inflammatory efficacy while minimizing side effects (33). Currently, nano-drug delivery systems have been widely applied in the treatment of sepsis (87, 133, 134). The objectives of these therapies are typically to reduce cell apoptosis, lower inflammatory factor levels, or ameliorate organ function injury, with the regulation of intestinal function often regarded as a concomitant beneficial effect. Stem cell therapy mainly utilizes the anti-inflammatory and immunomodulatory capabilities of mesenchymal stem cells to inhibit necroptosis and promote tissue repair (33). For example, adipose-derived mesenchymal stem cells not only ameliorate sepsis-induced acute lung injury but also modulate the abundance of gut microbiota (135). In addition, certain endogenous bioactive compounds also exert improving effects on intestinal function, such as lactoferrin (a multifunctional glycoprotein found in milk) and irisin (a myokine), which regulate gut microbiota composition and alleviate intestinal injury (86, 136).

Similar to nanotechnologies and stem cell therapies, the current evidence for most traditional Chinese medicines (TCM) in the treatment of sepsis remains limited to animal models and *in vitro* experiments, with their clinical translation still in its early stages. To date, only a few registered phase I/II clinical trials (e.g., the study on the Fuzheng Tongfu Jiedu Prescription (137) are ongoing. At the preclinical research level, current studies and applications mainly focus on three approaches: monomeric active compounds derived from animals or plants, single herbs or their complex extracts, and compound TCM formulas. In animal or cell experiments, the mechanisms of action of TCM in sepsis treatment primarily include regulation of gut microecology (138, 139) [such as increasing beneficial bacteria like Lachnospiraceae_NK4A136_group and suppressing harmful bacteria like Escherichia-Shigella (140)] and repair of the intestinal mucosal barrier (138, 141). Furthermore, animal studies suggest that TCM may suppress excessive inflammatory responses by modulating immune responses and cell signaling pathways, thereby achieving protection of the intestine and other organs. For example, hesperetin (38) attenuates neutrophil infiltration and the formation of neutrophil extracellular traps (NETs) ; Radix Pseudostellariae polysaccharides (138) inhibit the activation of the TLR4/MyD88/IKK/NF-κB signaling pathway; and Yazhicao (141) regulates the trimethylamine/trimethylamine N-oxide metabolic pathway.

It must be emphasized that the above mechanisms are all derived from preclinical studies. TCM have complex components, multiple targets, substantial batch-to-batch variability, and carry potential risks of herb-drug interactions and hepatotoxicity (142). Well-designed randomized controlled trials are needed in the future to validate their efficacy and safety.

## 8 Conclusion

In this review, a close bidirectional interaction between intestinal dysfunction and sepsis is discussed. The intestine not only serves as a crucial origin for the onset of sepsis but also acts as a major amplifier of its pathological progression. Besides, intestinal epithelial barrier dysfunction (particularly the impairment of tight junction proteins), gut microbiota imbalance, and the reduction of beneficial metabolites may together constitute the major mechanisms of gut-derived sepsis. Additionally, sepsis itself can further disrupt gut microbiota homeostasis and exacerbate intestinal permeability, potentially forming a vicious cycle. When intestinal barrier breakdown may lead to pathogen translocation, a systemic inflammatory response can be triggered, ultimately resulting in multiple organ dysfunction.

It is worth noting that the patient’s baseline intestinal functional status may significantly affects their prognosis, while conventional clinical supportive treatments (such as antibiotics, fluid resuscitation, and nutritional support) may inadvertently exacerbate intestinal injury while saving lives. Therefore, systematically assessing and protecting intestinal function is likely of great importance in the treatment of sepsis.

Currently, treatment strategies for sepsis have expanded from traditional immune regulation to increasingly encompass the restoration of intestinal microecology and barrier function. Although emerging research directions, such as microbiota-targeted biotherapeutics, interventions with microbial metabolites, and microbiota regulation by TCM, hold promising prospects, most of them remain at the basic or preclinical stage. Future efforts should be devoted to: (1) further elucidating the detailed pathological mechanisms of the intestine in sepsis; (2) exploring integrated therapeutic strategies that synergistically protect the intestine; and (3) promoting the clinical translation of precision interventions based on intestinal microecology, thereby potentially breaking the vicious cycle of intestinal injury-sepsis exacerbation and improving patient outcomes.
